# Omalizumab for severe allergic asthma: a life changing drug?

**DOI:** 10.1186/2045-7022-5-S2-P18

**Published:** 2015-03-23

**Authors:** Eris Mesonjesi, Erjola Piluri Ziu, Alfred Priftanji, Diana Qama

**Affiliations:** 1 University Hospital Center "Mother Teresa", Tirana, Albania; 2 American Hospital Tirana, Department of Allergology and Clinical Immunology, Tirana, Albania; 3 University Hospital Center "Mother Teresa", Department of Allergology and Clinical Immunology, Tirana, Albania; 4 Berat Hospital, Department of Allergology and Clinical Immunology, Berat, Albania

## Background

Asthma is an airway condition and is associated with significant morbidity and mortality. The recent updates for treatment of asthma recommends omalizumab for use as add-on therapy in adults and children >6 years of age with inadequately controlled severe persistent allergic asthma. Omalizumab, a humanized anti-IgE mAb, specifically binds free IgE. The clinical efficacy of omalizumab has been well documented in a number of clinical trials. In these studies omalizumab reduced exacerbations, asthma symptoms, inhaled corticosteroid and rescue medication use, and improved quality of life. Similar benefits have been reported in observational studies in "real-world" populations of patients. In our clinic we started recently using omalizumab in patients with severe allergic asthma using several biomarkers to monitor the asthma severity like spirometry, exhaled NO (FeNO), asthma control test (ACT), rhinitis control test. The data are still being collected since most of the patients are within the 16 week period of evaluation. We want to present one case from them that has completed the 16 week of treatment.

## Case report

A young woman 20 year-old, who suffered a lifetime history of severe allergic asthma since 3 years of age. She had several episodes of near fatal asthma the last one last winter. She is allergic to house dust mites, with continuous need for the rescue medication, and any kind of physical activity even walking for long distances is impossible for her. She couldn’t follow the College because of the disease. Omalizumab was started on June 2014, 150 mg every 4 weeks, as add on therapy to fluticasone-salmeterol 250/50mcg. On week 16 she had better scores in different biomarkers: ACT: 17 from 15 on day 0, spirometry: FEV1 1.62 (57%) on day 0 to 1.77 (64.5%) on week 16, and FeNO: 16 ppb on day 0 to 14ppb on week 16. The patient is feeling much better; she had no exacerbation and no need for the rescue medication. She is able to walk for the first time long distances without having an asthma attack.

## Conclusion

Omalizumab is effective in reducing asthma exacerbations and hospitalisations as an adjunctive therapy to inhaled steroids. Omalizumab is found to be generally safe and well-tolerated. Our patient had no side effects. We observed small changes in the biomarkers but the therapy looks very promising for the future.

## Consent

Written informed consent was obtained from the patient for publication of this abstract and any accompanying images. A copy of the written consent is available for review by the Editor of this journal.

